# Susceptibility of Chickens to Low Pathogenic Avian Influenza (LPAI) Viruses of Wild Bird– and Poultry–Associated Subtypes

**DOI:** 10.3390/v11111010

**Published:** 2019-10-31

**Authors:** Saskia A. Bergervoet, Evelien A. Germeraad, Marc Alders, Marit M. Roose, Marc Y. Engelsma, Rene Heutink, Ruth Bouwstra, Ron A.M. Fouchier, Nancy Beerens

**Affiliations:** 1 Department of Virology, Wageningen Bioveterinary Research, 8200 AB Lelystad, The Netherlands; saskia.bergervoet@wur.nl (S.A.B.); evelien.germeraad@wur.nl (E.A.G.); marc.alders@wur.nl (M.A.); marit.roose@wur.nl (M.M.R.); marc.engelsma@wur.nl (M.Y.E.); rene.heutink@wur.nl (R.H.); 2 Department of Viroscience, Erasmus MC, 3015 GD Rotterdam, The Netherlands; r.fouchier@erasmusmc.nl; 3 GD Animal Health Service, 7418 EZ Deventer, The Netherlands; r.bouwstra@gddiergezondheid.nl

**Keywords:** avian influenza virus, low pathogenic avian influenza, wild birds, poultry, chickens, shedding, innate immune response

## Abstract

Analysis of low pathogenic avian influenza (LPAI) viruses circulating in the Netherlands in a previous study revealed associations of specific hemagglutinin (HA) and neuraminidase (NA) subtypes with wild bird or poultry hosts. In this study, we identified putative host associations in LPAI virus internal proteins. We show that LPAI viruses isolated from poultry more frequently carried the allele A variant of the nonstructural protein (NS) gene, compared to wild bird viruses. We determined the susceptibility of chickens to wild bird–associated subtypes H3N8 and H4N6 and poultry-associated subtypes H8N4 and H9N2, carrying either NS allele A or B, in an infection experiment. We observed variations in virus shedding and replication patterns, however, these did not correlate with the predicted wild bird- or poultry-associations of the viruses. The experiment demonstrated that LPAI viruses of wild bird-associated subtypes can replicate in chickens after experimental infection, despite their infrequent detection in poultry. Although the NS1 protein is known to play a role in immune modulation, no differences were detected in the limited innate immune response to LPAI virus infection. This study contributes to a better understanding of the infection dynamics of LPAI viruses in chickens.

## 1. Introduction

Avian influenza (AI) viruses are influenza A viruses that circulate among a broad range of wild bird species, particularly birds of the orders *Anseriformes* (ducks, geese, swans) and *Charadriiformes* (gulls, terns, waders) [1], and can also infect domestic poultry. AI viruses are divided into subtypes based on the surface glycoproteins hemagglutinin (HA) and neuraminidase (NA), which are involved in the attachment and release from host cells [2], respectively. Most viruses are low pathogenic avian influenza (LPAI) viruses that produce subclinical infections in poultry or, occasionally, cause mild respiratory disease, a reduction in egg production and low mortality [3]. LPAI viruses of HA subtypes H5 and H7 can mutate into highly pathogenic avian influenza (HPAI) variants that can cause severe progressive disease and high mortality in birds [4]. Outbreaks of HPAI viruses can have serious impact on animal health and economic consequences for the commercial poultry industries. In addition, some strains can be transmitted to humans [5], causing major concern for public health worldwide.

In birds, 16 HA subtypes (H1–H16) and 9 NA (N1–N9) subtypes have been found in numerous combinations [6,7]. Some of these subtypes are specific to certain avian species, of which H13 and H16 viruses in gulls is a prime example [7]. Our previous study on LPAI viruses circulating in wild birds and poultry in the Netherlands also revealed close host-dependent associations among other HA and NA subtypes [8]. During a 10-year surveillance period in the Netherlands, LPAI viruses of subtypes H3N8 and H4N6 were predominantly detected in wild ducks, in particular mallards, but rarely detected in poultry. In contrast, LPAI viruses of subtypes H8N4 and H9N2 were most frequently detected in Dutch poultry but only sporadically detected in wild birds. These findings suggest that transmission to poultry is selective and is likely influenced by viral factors that determine host range.

Besides HA and NA, the viral genome encodes for internal proteins including polymerase basic protein 1 (PB1), polymerase basic protein 2 (PB2), polymerase acidic protein (PA), matrix proteins (M1 and M2), nucleoprotein (NP), and nonstructural proteins (NS1 and NS2). The NS proteins circulate in two lineages (NS allele A and B) that differ by around 30% of their amino acids [9]. The internal proteins are responsible for viral functions, such as genome replication and expression and virus assembly [10]. The NS1 protein can modulate the host immune response and protein expression, and thereby acts as an important virulence factor [11]. Several of the internal proteins have been described to influence the infectivity, pathogenicity and transmissibility of AI viruses in a host-dependent manner [12], but their role in host-dependent transmission between avian species is largely unknown. Increased knowledge on the ability of different AI virus subtypes and genotypes to infect poultry would contribute to a better understanding of virus epidemiology. In addition, the identification of viral factors that influence host range can be used for predicting the risk of infection in poultry and can help to design more efficient surveillance programs for the early detection of potentially dangerous strains.

In this study, we analyzed the association of LPAI virus internal proteins with wild bird or poultry hosts. We show that LPAI viruses isolated from poultry more frequently contain NS allele A, compared to wild bird viruses. The susceptibility of chickens to wild duck-origin LPAI viruses of wild bird-associated subtypes H3N8 and H4N6 and poultry-associated subtypes H8N4 and H9N2, carrying either NS allele A or B, was studied in an infection experiment. We analyzed the pattern and route of viral shedding, which not only provides information on potential replication sites but also gives an indication of the ability of virus transmission to other birds. In addition, we analyzed virus replication and immunological responses in the respiratory and intestinal organs, which are main target tissues of LPAI viruses. This study contributes to a better understanding of the infection dynamics of different LPAI virus strains in chickens, which can be used to improve current surveillance programs.

## 2. Material and Methods

### 2.1. Ethical Statement

The animal experiment and associated procedures were in accordance with the national regulations on animal experimentation and the project license was approved by the Dutch Central Authority for Scientific Procedures on Animals (CCD) (permit number ADV401002015317; experiment number 2016.D-0057.001). The animal procedures were performed conforming to the guidelines from the European Union directive 2010/63/EU of 22 September 2010 on the protection of animals used for scientific purposes [13].

### 2.2. Virus Selection

The subtypes tested in this study were selected based on their association with wild birds or poultry as observed in a previous surveillance study in the Netherlands [8]. To identify host-associated features in the internal proteins, a statistical comparative analysis was performed using the metadata-driven comparative analysis tool for sequences (meta-CATS) of the Influenza Research Database (IRD) (https://www.fludb.org) [14]. For this analysis, we used the amino acid (aa) sequences of the internal proteins (PB1, PB2, PA, M1, M2, NP, NS1, and NS2) of the same set of 162 wild bird viruses and 42 poultry viruses as analyzed previously [8]. For each protein, a multiple sequence alignment was generated in MUSCLE version 3.8.31 [15]. Subsequently, the alignments were submitted to the automated meta-CATS pipeline in two groups according to their host source (i.e., wild bird or poultry). In the statistical tool, a chi-square test of independence was performed at each aa position to identify residues that significantly differed between the groups (*p* < 0.05).

### 2.3. Virus Isolation and Propagation

The viruses used in this study were provided by Erasmus MC (Rotterdam, the Netherlands) (Table S1). The viruses were initially isolated from oropharyngeal (OP) or cloacal (CL) swabs collected from wild ducks as part of the national AI virus surveillance program in wild birds in the Netherlands. The whole genome sequences were generated in a previous study [8]. Virus stocks were generated by two passages in 9–11-day-old specific pathogen free (SPF) embryonated chicken eggs (ECEs). The virus stocks were titrated using standard methods to determine the median egg infectious dose (EID_50_) titres [16]. The median tissue culture infective dose (TCID_50_) titres were determined by end-point titration in Madin-Darby Canine Kidney (MDCK) cells, as described previously [17]. The virus stocks were diluted in sterile phosphate buffered saline (PBS) immediately prior to use in order to obtain 10^6^ EID_50_/mL inoculum.

### 2.4. Animals and Housing

A total of 184 six-week-old SPF White Leghorn chickens (*Gallus gallus domesticus*) of both sexes were obtained from MSD Animal Health (Boxmeer, the Netherlands). White Leghorn chickens were chosen for this research as it represents the most common and economically important poultry type in the Netherlands [18]. The experiment was performed in biosafety level 2 (BSL 2) facilities at Wageningen Bioveterinary Research (WBVR, Lelystad, the Netherlands). The chickens were housed in temperature-controlled rooms under optimal light conditions and humidity, and feed and water were provided ad libitum. Each experimental group was housed separately in floor pens with solid livestock dividing panels, and personnel changed clothes to prevent cross-contamination between groups.

### 2.5. Experimental Design

**Figure 1:**
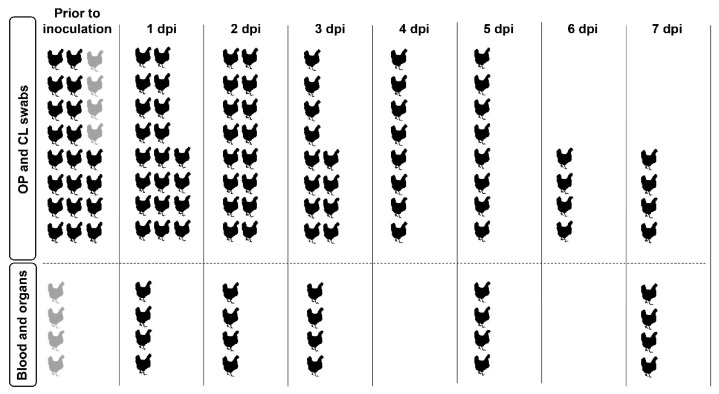
Sample collection per experimental group. A total of 184 six-week-old specific pathogen free (SPF) White Leghorn chickens were divided into eight experimental groups of 20 chickens (virus-inoculated groups) and one experimental group of 24 chickens (control group). Prior to inoculation, oropharyngeal (OP) and cloacal (CL) swabs were collected from all chickens of each experimental group to confirm the absence of current AI virus infection. In addition, blood and organs were collected from four chickens in the control group (shown in grey). After inoculation, OP and CL swabs were taken from live birds daily to seven days post inoculation (dpi) to determine viral shedding. Four chickens from each experimental group were euthanized at 1, 2, 3, 5, and 7 dpi to collect blood and organs. The experiment was terminated at 7 dpi.

The chickens were randomly divided into eight experimental groups (virus-inoculated groups) of 20 chickens and one experimental group (control group) of 24 chickens (Figure 1). Individual birds of each experimental group were numbered randomly to select chickens for tissue collection at different time points. At day 0, prior to inoculation, OP and CL swabs were collected from all chickens to confirm the absence of current AI virus infection. In addition, blood samples were collected by heart puncture from the control group (*n* = 4) under anaesthesia by intramuscular (IM) administration of 0.4 mL Xylazine and Ketamine mixture. After blood collection, the chickens were immediately euthanized by intravenous (IV) administration of 2.0 mL Euthasol 50% solution (AST Farma, Oudewater, the Netherlands), and tissue samples of various organs were collected, including the trachea, lung, and ileum.

Inoculation was performed via intranasal (IN) and intratracheal (IT) administration of 0.1 mL of 10^6^ EID_50_/mL inoculum per route (inoculation dose of 10^5.3^ EID_50_ per bird). The inoculation dose was confirmed by back-titration on MDCK cells and conversion of the TCID_50_ titres into equivalent EID_50_ titres. For each experimental group, the viral dose was within the range of 0.5 log10 EID_50_/mL of the target titre. All birds were observed daily for clinical signs of disease. OP and CL swabs were taken for virus detection from live birds daily to seven days post inoculation (dpi) to determine viral shedding. Four chickens from each experimental group were euthanized, using the same method as the control group, at 1, 2, 3, 5, and 7 dpi to collect blood and organs. The experiment was terminated at 7 dpi.

### 2.6. Antibody Detection

Blood samples were left overnight at RT for serum separation [19], and serum samples were stored at −20 °C until testing. For influenza virus-specific antibody detection, serum was tested by anti-NP ELISA (FlockChek AI MultiS-Screen Ab Test Kit (IDEXX Europe B.V., Hoofddorp, the Netherlands)) according to the manufacturers’ instructions. Hemagglutination inhibition (HI) testing for HA subtype-specific antibodies was done using standardized homologous H3, H4, H8, and H9 antigens. HI test results were reported as log_2_ HI titres, with titres of 3 log_2_ or higher considered positive.

### 2.7. Virus Detection in Swabs

Swabs were placed in 2.0 mL Tryptose Phosphate Broth 2.95% containing gentamicin and stored at −80 °C until testing. For virus detection, total RNA was extracted from the swab specimens using the MagNA Pure 96 system (Roche, Basel, Switzerland) with the MagNA Pure 96 DNA and Viral NA Small Volume Kit (Roche). Influenza A virus was detected by a quantitative real-time reverse transcription polymerase chain reaction targeting the matrix gene (M-PCR), as described previously [20]. For each virus, a standard curve for virus quantification was taken along that consisted of ten-fold serial dilutions of the working stocks with a known EID_50_ titre. The standard curve was used to convert the Ct values into equivalent EID_50_ titres and determine PCR efficiency. Results were reported as mean equivalent log10 EID_50_/mL titres and their standard deviation (SD) with a lower detection limit of 10^1.7^ EID_50_/mL and plotted using Graphpad PRISM 8. Chickens were considered positive for viral shedding if virus was detected in swab samples at any time during the experiment.

### 2.8. Virus Detection in Tissue

Tissues collected for virus detection were snap-frozen on dry ice and stored at −80 °C until processing. The frozen tissue samples (100 mg) were homogenised in 1.0 mL Trizol (TRI Reagent (Thermo Fisher Scientific, Waltham, MA, USA)) [21]. Isolation of total RNA from the homogenized tissue was performed using the Direct-Zol RNA Microprep isolation kit (Zymo Research, Irvine, CA, USA) with DNAse treatment according to the manufacturers’ protocol. An M-PCR was performed to quantitate viral RNA in the tissue samples, as described previously [20]. Results were reported as mean Ct values and their SD.

### 2.9. Cytokine Expression in Tissue

To measure cytokine expression, the quantity of the extracted RNA from tissue samples was assessed using the Nanodrop spectrophotometer (Thermo Fisher Scientific). RNA quality was inferred using the 2100 Bioanalyzer system (Agilent Technologies, Santa Clara, CA, USA). Samples meeting a RNA integrity number (RIN) score of 6.5 or higher have been classified as high-quality RNA samples obtained from frozen tissue [22], and were therefore included in this study. RNA (200 ng) was converted into cDNA using the Superscript IV First-Strand Synthesis System (Thermo Fisher Scientific). Quantitative real-time PCR was performed using PowerUp SYBR Green Master Mix (Thermo Fisher Scientific) in the ABI 7500 Real-Time PCR system (Thermo Fisher Scientific) [21].

A panel of five candidate reference genes in chickens (HPRT1, RPLP0, HMBS, TBP, RPL13) was selected based on literature [23] and tested using the extracted RNA samples from tissues of seven birds from the virus-inoculated and control group. From this panel, two stably expressed reference genes (RPLP0 and TBP) were selected for each organ separately using NormFinder [24]. Cytokine and TLR mRNA expression were measured by a PCR targeting IL-6, IL-1β, IFN-α, IFN-β, and TLR7 mRNAs. The primer sets used were identical to those described previously [21], except that the IL-1β forward primer was replaced with 5′-CAGCAGCCTCAGCGAAGAG-3′. A standard curve of a plasmid containing the gene of interest (GenScript Biotech, Piscataway, NJ, USA) was used to determine PCR efficiency. Results were reported as fold change in mRNA expression relative to the control group and their SD calculated using the ΔΔ*C*_t_ method (2^−ΔΔ*C*t^) [25]. Comparison between virus-inoculated and control groups was performed using the unpaired, two-tailed Student’s *t*-test, with significance defined as *p* < 0.05. The statistical analyses were conducted using Graphpad PRISM 8.

### 2.10. Cluster Analysis of Internal Genes

We performed genetic cluster analysis based on the internal genes (PB1, PB2, PA, NP, MP, and NS) to determine if genes similar to those of the inoculated viruses have previously been isolated from poultry. For this analysis, we used a dataset of publicly available sequences of around 20,000 avian-origin AI viruses obtained from GISAID’s EpiFlu database (http://www.gisaid.org) [26] on 27 May 2019. For each internal gene, the sequences were aligned in MAFFT version 7.427 [27,28] and curated in Aliview version 1.26 [29]. Partial sequences and sequences containing multiple ambiguous bases (Ns) were excluded from the analysis. The remaining sequences were clustered against the sequences of the inoculated viruses in CD-HIT-EST-2D [30,31]. A sequence identity threshold value of 1.5% was used to generate clusters of sequences that share nucleotide sequence identities of at least 98.5% with the inoculated virus. For each cluster, the total number of sequences (cluster size) and the number of sequences derived from poultry were determined.

## 3. Results

### 3.1. Identification of Host-Associations in the Internal Proteins

To identify amino acid (aa) residues in the internal proteins that significantly differ between viruses isolated from wild birds and poultry, we applied meta-CATS analysis using the sequences of 162 wild bird viruses and 42 poultry viruses isolated in the Netherlands. The chi-squared analysis identified 135 statistically significant aa residues that differed between the host groups (*p* < 0.05). A total of 82 aa residues were located in the NS proteins due to the presence of two distinct NS alleles (NS allele A and B) (Table S2). Viruses isolated from poultry contained relatively more frequently NS allele A (83%) than viruses isolated from wild birds (62%). This skewed distribution may suggest that poultry is more prone to infection with LPAI virus strains carrying NS allele A.

We identified 53 statistically significant aa residues that differed between wild bird and poultry viruses but were not linked to the NS alleles. These residues were located in PB1 (10 of 48), PB2 (11 of 53), PA (14 of 53), NP (11 of 53), M1 (1 of 53), M2 (1 of 53), NS1 (4 of 53), and NS2 (1 of 53). A total of 44 residues were present in more than 90% of the viruses in both host groups, and four residues were located at highly variable aa positions, as three or more aa variants were detected at these positions. The five aa residues for which larger differences in occurrence between wild bird and poultry viruses were found are listed in Table S2. These were mostly arginine (R) to lysine (K) substitutions, with unknown effect on protein function. Based on the suspected host-association of the NS protein, we selected LPAI virus strains of NS allele A and B for further evaluation in the animal experiment.

### 3.2. Clinical Signs and Seroconversion in LPAI Infected Chickens

Six-week-old SPF chickens were inoculated with eight wild duck-origin LPAI viruses of wild bird-associated (H3N8 and H4N6) and poultry-associated (H8N4 and H9N2) subtypes, with either NS allele A or B, to study their susceptibility to infection (Table S1). The chickens were examined for clinical signs, swabbed daily, and euthanized at selected time points to collect organs and blood (Figure 1).

During the seven-day experiment, no obvious clinical signs and no mortality were observed. There were no antibodies detected in serum of the control chickens. Influenza A virus NP-specific antibodies were detected by ELISA in chickens inoculated with H8N4 NS A virus (four of four chickens) and H9N2 NS A virus (one of four chickens) at 5 dpi (Table S3), indicating rapid initiation of antibody production in these chickens. At 7 dpi, seroconversion was observed in chickens from all experimental groups, with the exception of chickens inoculated with H8N4 NS B virus. HA subtype-specific antibodies were detected upon inoculation with H8N4 NS A virus at 5 dpi in three of four chickens (mean HI titre of 4.3 ± 0.6 log_2_) and at 7 dpi in two of four chickens (HI titres of 5 log_2_). In the groups inoculated with H3N8 NS A and B viruses, subtype-specific antibodies were detected at 7 dpi in one of four chickens (HI titres of 3 log_2_).

### 3.3. Viral shedding of LPAI Infected Chickens

**Figure 2:**
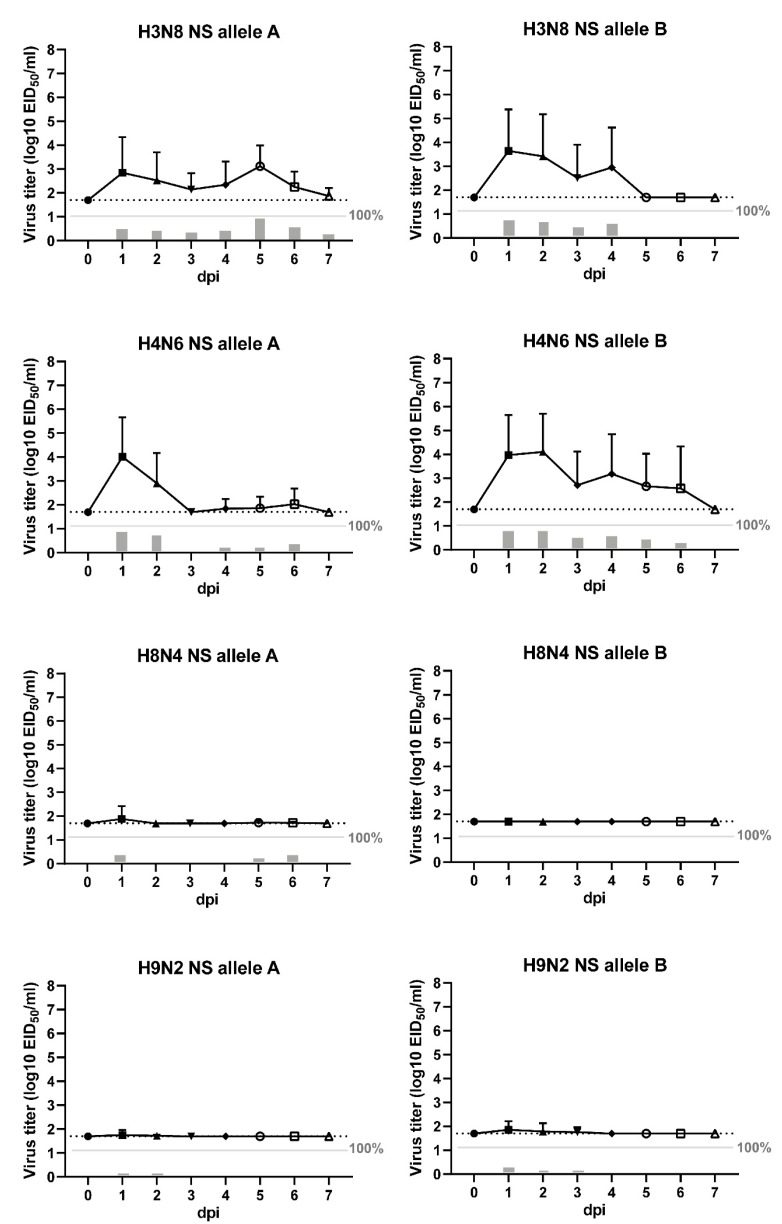
Oropharyngeal shedding. Virus detected in oropharyngeal (OP) swabs collected from chickens inoculated by the intranasal (IN) and intratracheal (IT) route with eight strains of low pathogenic avian influenza (LPAI) viruses (10^5.3^ median egg infectious dose (EID_50_) per bird). The swabs were taken daily from live birds to seven days post inoculation (dpi) for virus detection by influenza virus-specific PCR (M-PCR). Viral shedding is expressed as the mean equivalent log10 EID_50_/mL titre ± standard deviation (SD) with a lower detection limit of 10^1.7^ EID_50_/mL (dashed line). The grey bars below the solid line indicate the percentage of positive swabs.

**Figure 3:**
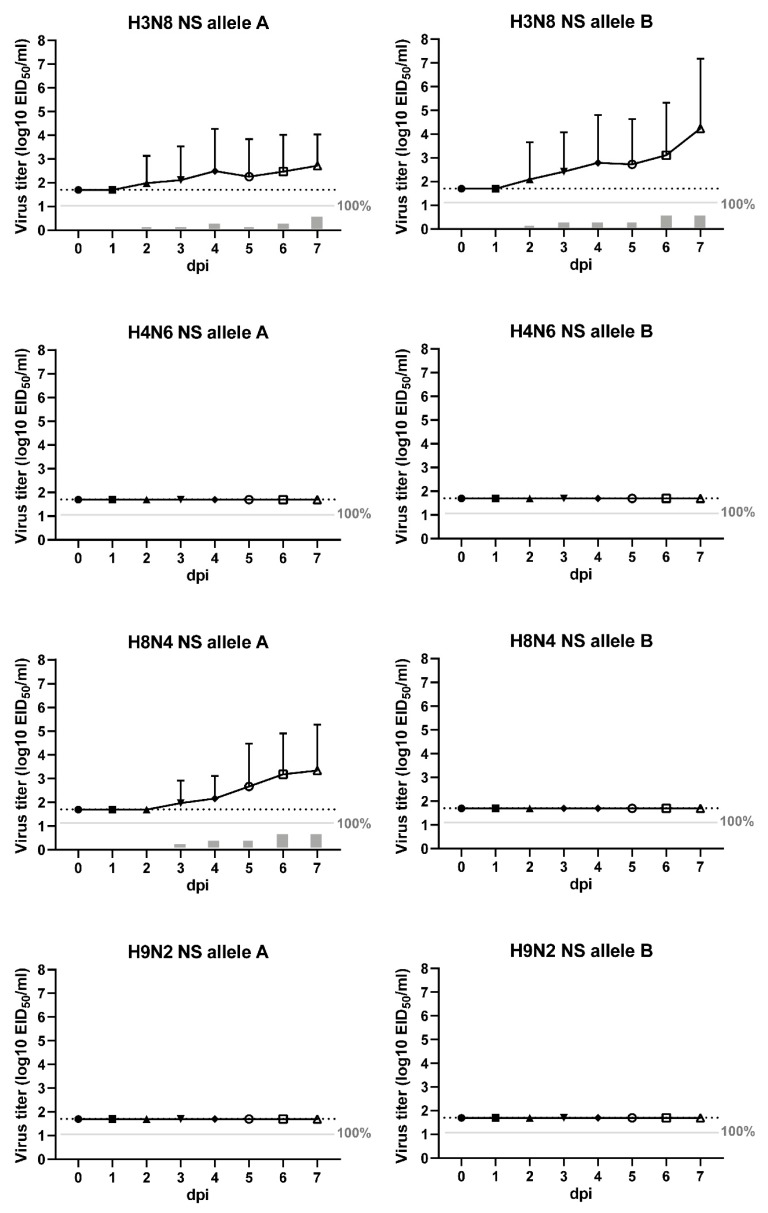
Cloacal shedding. Virus detected in cloacal (CL) swabs collected from chickens inoculated by the intranasal (IN) and intratracheal (IT) route with eight strains of low pathogenic avian influenza (LPAI) viruses (10^5.3^ median egg infectious dose (EID_50_) per bird). The swabs were taken daily from live birds to seven days post inoculation (dpi) for virus detection by influenza virus-specific PCR (M-PCR). Viral shedding is expressed as the mean equivalent log10 EID_50_/mL titre ± standard deviation (SD) with a lower detection limit of 10^1.7^ EID_50_/mL (dashed line). The grey bars below the solid line indicate the percentage of positive swabs.

Viral shedding upon inoculation of the LPAI viruses was measured daily by the detection of viral RNA in swab samples using influenza virus-specific M-PCR. The mean virus titres in oropharyngeal (OP) and cloacal (CL) swabs for each experimental group are plotted over time in Figure 2 and Figure 3, respectively. The number of birds shedding virus and the mean shedding titres are provided in Tables S4 and S5. No virus was detected in swab samples collected from the control birds. Viral shedding was observed in all virus-inoculated groups, except in chickens inoculated with H8N4 NS B virus.

Shedding through the oropharyngeal route was predominantly detected in chickens inoculated with H3N8 NS A virus (12 of 20 chickens), H3N8 NS B virus (17 of 20 chickens), H4N6 NS A virus (19 of 20 chickens), and H4N6 NS B virus (16 of 20 chickens). The mean onset of OP shedding was 1.4 ± 1.1 dpi. In chickens inoculated with H3N8 NS A virus, a peak of shedding was observed at 1 and 5 dpi, with a mean shedding titre of 10^3.8^ EID_50_. In this group, one chicken was tested positive for OP shedding at 7 dpi, although the virus titre was low (10^2.4^ EID_50_). No OP shedding was observed at 7 dpi in other experimental groups. In chickens inoculated with H3N8 NS B virus, a peak of shedding was observed at 1–2 dpi, with a mean shedding titre of 10^4.7^ EID_50_, which declined and resolved by 5 dpi. H4N6 NS A virus-infected chickens showed a peak of shedding at 1 dpi (mean shedding titre of 10^4.8^ EID_50_), followed by a fast decline to low virus titres between 3–7 dpi. Chickens inoculated with H4N6 NS B virus showed a peak of OP shedding at 1–2 dpi, with a mean shedding titre of 10^4.8^ EID_50_, which declined and resolved by 7 dpi. However, limited shedding was detected in chickens inoculated with H8N4 NS A virus (7 of 20 chickens), H9N2 NS A virus (4 of 20 chickens), and H9N2 NS B virus (6 of 20 chickens), and no OP shedding was detected in chickens inoculated with H8N4 NS B virus (0 of 20 chickens). 

In addition, limited shedding through the cloacal route was observed in chickens inoculated with H3N8 NS A virus (2 of 20 chickens), H3N8 NS B virus (7 of 20 chickens), and H8N4 NS A virus (3 of 20 chickens). The majority of these chickens (11 of 12 chickens) were also positive for OP shedding. The mean onset of CL shedding was 3.9 ± 1.5 dpi. The mean virus titre was highest at 7 dpi in all three experimental groups, with mean shedding titres ranging between 10^3.7–6.8^ EID_50_. No virus was detected in the CL swabs of chickens inoculated with both H4N6 viruses, H8N4 NS B virus and both H9N2 viruses. The results demonstrate variations in viral shedding patterns and routes between LPAI viruses of wild bird– and poultry-associated subtypes and NS alleles in chickens. 

### 3.4. Viral Replication in the Respiratory and Intestinal Tract

Tissues collected from the trachea, lung and ileum were tested by M-PCR to determine viral replication in the respiratory and intestinal tract of four chickens from each experimental group (Table 1). No virus was detected in tissues collected from the control chickens At 1 dpi, virus was detected in trachea and lung tissues of chickens from all virus-inoculated groups, indicative of local virus replication. Remarkably, virus was detected in trachea or lung of all chickens inoculated with H8N4 NS A virus and three of four chickens inoculated with H9N2 NS B virus, despite the fact that OP shedding was limited in these groups. At 3 and 5 dpi, most tissues examined were tested virus-negative, indicating a short persistence of LPAI virus in the chicken respiratory tract. At 7 dpi, virus was detected in the ileum collected from chickens inoculated with H3N8 NS A (one of four chickens), H3N8 NS B (two of four chickens) and H8N4 NS A (all four chickens) viruses, which were also positive for CL shedding. This shows that LPAI virus replication in the intestine and subsequent excretion via the cloaca is strain-dependent. Overall, virus was mainly detected in the respiratory tissues at 1 dpi and intestinal tissues at 7 dpi, which correlates with the pattern of viral shedding over time.

### 3.5. Cytokine mRNA Expression in the Trachea and Ileum

**Figure 4:**
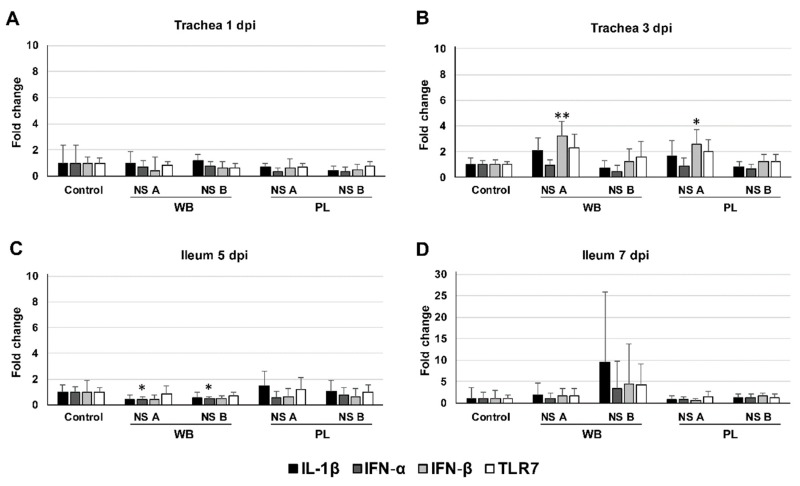
Cytokine mRNA expression in the trachea and ileum. Fold change in IL-1β, IFN-α, IFN-β, and TLR7 mRNA levels in trachea at (**A**) day 1 and (**B**) day 3, and in ileum at (**C**) day 5 and (**D**) day 7 post inoculation (dpi) of chickens with low pathogenic avian influenza (LPAI) viruses of wild bird-associated H3N8 and H4N6 (WB) and poultry-associated subtypes H8N4 and H9N2 (PL) carrying nonstructural protein (NS) allele A or B. Results of two experimental groups were pooled into a single representative group as follows: H3N8 and H4N6 viruses of NS allele A (WB/NS A), H3N8 and H4N6 viruses of NS allele B (WB/NS B), H8N4 and H9N2 viruses of NS allele A (PL/NS A), and H8N4 and H9N2 viruses of NS allele B (PL/NS B). mRNA expression is shown as the mean fold change relative to the control group ± standard deviation (SD). * *p* < 0.05, ** *p* < 0.01.

For analysis of the inflammatory cytokine response, we measured the levels of IL-6, IL-1β, IFN-α, IFN-β, and TLR7 mRNA in tissues collected from the trachea and ileum by quantitative PCR (Figure 4). The measured expression levels were normalized using a selected panel of reference genes. We determined the mean fold change in mRNA expression in chickens infected with LPAI viruses of wild bird-associated (H3N8 and H4N6) and poultry-associated (H8N4 and H9N2) subtypes, carrying either NS allele A or B, relative to uninfected chickens in the control group. Different time points were selected for the trachea (1 and 3 dpi) and ileum (5 and 7 dpi) based on the detection of virus in swabs and organs. Levels of IL-6 mRNA were almost undetectable in all tissues examined, and therefore excluded for analysis. 

In the trachea, no significant change in cytokine expression was observed at 1 dpi. At 3 dpi, levels of IFN-β mRNA were slightly induced in chickens inoculated with the NS allele A variants of wild bird-associated and poultry-associated subtypes (mean fold induction of 3.2 ± 1.1 and 2.6 ± 1.1, respectively). In the ileum, slightly reduced levels of IFN-α mRNA were measured at 5 dpi in chickens inoculated with wild bird-associated subtypes of NS allele A and B (mean fold reduction of 2.3 ± 0.2 and 2.1 ± 0.1, respectively). At 7 dpi, we observed increased within-group variability in cytokine mRNA levels in the ileum of chickens inoculated with wild bird-associated subtypes of NS allele B. This suggests that these strains led to changes in cytokine expression in a subset of the infected chickens. However, overall, the results indicate that LPAI viruses elicit limited innate immune responses in chickens.

### 3.6. Genetic Cluster Analysis of the Inoculated Viruses

The susceptibility of chickens to the LPAI viruses of specific subtypes or NS alleles was not consistent with their frequency of isolation in the field. We therefore performed genetic cluster analysis for the internal genes (PB1, PB2, PA, NP, MP, and NS) to determine if genes similar to those of the inoculated viruses have previously been isolated from poultry. This may reveal genetic links between the inoculated viruses and poultry hosts that can help to explain the unexpected results of the infection experiment. For this analysis, the full-length nucleotide sequences of the internal genes were compared with those deposited in online databases. Genes similar to those of the inoculated viruses were identified by genetic cluster analysis using a sequence identity threshold value of 1.5%. For each genetic cluster, the cluster size and the number of viruses isolated from poultry were determined (Table 2).

The genetic cluster analysis confirmed the association of NS allele A with poultry, as viruses carrying NS allele A clustered more often with poultry viruses compared to viruses carrying NS allele B. Interestingly, one or more gene segments encoding for the viral polymerase subunits (PB1, PB2, and PA) clustered with poultry viruses for both H3N8 viruses, both H4N6 viruses and H8N4 NS A virus, which could also replicate in chickens. In contrast, H8N4 NS B virus and both H9N2 viruses, which did not replicate in chickens, contained polymerase gene segments that were restricted to wild bird isolates. These findings suggest that the polymerase complex may play a role in infection of chickens and may be involved in the transmissibility of LPAI viruses between avian hosts.

## 4. Discussion

In a previous study on LPAI viruses circulating in wild birds and poultry in the Netherlands, we found close host-dependent associations among HA and NA subtypes, suggesting selective virus transmission to poultry [8]. Viruses of subtypes H3N8 and H4N6 were found to be associated with wild birds, whereas H8N4 and H9N2 were found to be associated with poultry. In the present study, we examined these host-subtype associations in vivo. Chickens were inoculated with eight strains of wild duck-origin LPAI viruses of wild bird- and poultry-associated subtypes. No clinical signs were observed during the experiment, which is consistent with the low pathogenicity of the viruses. The absence of antibody responses in most virus-inoculated chickens may be due to the short duration of the experiment. Viral shedding was observed in 82 of 160 virus-inoculated chickens, predominantly via the OP route early after inoculation, which is consistent with previous studies [32,33,34]. Evidence of viral replication was found in all virus-inoculated groups, but strain-dependent variations in susceptibility and shedding patterns were observed between groups.

Contrary to our expectations based on field observations, LPAI viruses of wild bird-associated subtypes H3N8 and H4N6 replicated in chickens. Most chickens inoculated with the H3N8 viruses shed virus through both the OP and CL route. In contrast, the H4N6 viruses were exclusively shed through the OP route, implying that the H4N6 viruses used in this study replicate more efficiently in the respiratory tract than in the intestinal tract. Previous studies have shown that Asian H3N8 and H4N6 strains of wild bird-origin are able to infect chickens, although replication efficiency strongly differed between strains [35,36,37]. In Europe, recent outbreaks of H3N1 viruses in Belgium have also demonstrated the ability of H3 viruses to infect chickens [38]. Phylogenetically, the HA gene of the H3N1 virus clusters with other Eurasian strains of different subtype combinations and internal gene compositions, but no more than 98.3% nucleotide sequence identity was found by BLAST (results not shown). The results in this study demonstrate that chickens are experimentally susceptible for H3N8 and H4N6 viruses that have been circulating in Europe, suggesting that these viruses can also be transmitted from anseriform to galliform hosts. Nevertheless, these subtypes were rarely detected in chickens during a ten-year surveillance period in the Netherlands [8]. Of the LPAI virus subtypes H8N4 and H9N2 that were frequently found in poultry, only one H8N4 strain replicated efficiently in chickens. The H8N4 virus carrying NS allele A was shed in high concentrations through the cloaca, replicated in the respiratory and intestinal tract, and induced a more rapid seroconversion in chickens compared to the other inoculated viruses. Despite the frequent detection of H8N4 and H9N2 subtypes in poultry during the ten-year surveillance period, no to limited virus was detected in the swabs or tissues of chickens inoculated with the other H8N4 and H9N2 strains, indicating low susceptibility for these strains. 

Several factors may have contributed to these unexpected results. One explanation could be that H3N8 and H4N6 viruses have been introduced into poultry but have not been detected during surveillance. This is considered unlikely, as poultry flocks are screened at least once a year for the presence of antibodies against AI viruses, which are generally detectable up to several weeks or months after infection [39]. However, the antibody responses may differ in duration and intensity between virus subtypes and strains. H3N8 and H4N6 viruses may cause shorter antibody responses in chickens compared to other subtypes, resulting in a less frequent detection in poultry. Analysis of humoral responses to diverse LPAI viruses in chickens could provide more insight in variations in the duration and intensity of antibody responses between subtypes. Furthermore, the fact that the LPAI viruses used in this study originate from wild ducks, and may have been adapted to anseriform hosts, could have contributed to the inefficient replication of most H8N4 and H9N2 strains in chickens. The H9N2 viruses used in this study are not related to the poultry-adapted H9N2 viruses that have circulated enzootically in poultry in Asia, North Africa, and the Middle East, with sporadic spillovers to humans (results not shown). Finally, discrepancies between experimental studies and field observations may be due to environmental conditions, such as the number and density of chickens, virus inoculation dose and route, and concurrent infections, which can influence virus infection dynamics.

Alternatively, poultry have not been exposed to the LPAI viruses that were detected in wild birds. The fact that H3N8 and H4N6 viruses have frequently been isolated from cloacal swabs of wild birds indicates the possibility of viral exposure of poultry via faecal droppings. Therefore, these LPAI viruses may circulate in wild bird species that have not been in contact with poultry. Wild bird surveillance in the Netherlands is strongly biased towards mallards [8], while these may not be risk species for AI virus introduction into poultry. This is supported by previous genetic analysis that showed no direct relationship between wild bird and poultry viruses [8]. The biased surveillance in wild birds may also explain why H8N4 and H9N2 viruses were less often detected in wild birds. Possibly these viruses predominantly circulate among other understudied wild bird species. An important limitation is that wild bird surveillance is solely based on virological monitoring. In contrast to antibody detection, virus can only be detected during a relatively short time frame. A previous surveillance study in Sweden reported the detection of H8 and H9 viruses during late spring and early summer, outside the prevalence peak of AI viruses in wild birds [40]. In this period, the sampling frequency of wild birds in the Netherlands is low, which may explain the limited detection of H8N4 and H9N2 viruses. 

In this study, we also examined host-associations in genes encoding for internal proteins. This analysis revealed that NS allele A—although predominant in both wild birds and poultry—was relatively more frequently detected in poultry compared to wild birds. A similar distribution was observed among online available sequences, in which viruses collected from galliform species carried more often NS allele A compared to viruses collected from anseriform species [41]. The skewed distribution suggests that NS A viruses may have an increased ability to infect poultry. Previous studies have shown that the NS1 protein can influence viral replication across hosts and determines viral fitness and pathogenicity in chickens by evading the immune response through inhibition of IFN type I production [42,43,44]. The distinct NS alleles have also been associated with host-specific transmission of AI viruses based on in vitro studies using avian and mammalian cells. In mammalian cells, NS A viruses were able to suppress IFN type I production more efficiently compared to NS B viruses [45,46]. In avian cells, NS A viruses replicated more efficiently in chicken and turkey cells, whereas NS B viruses replicated more efficiently in duck cells [47], suggesting that the NS alleles can also influence virus transmission at the wild bird–poultry interface.

Our study shows that LPAI viruses of both NS alleles were able to induce viral shedding and replicate in respiratory and intestinal organs of chickens. This demonstrates that the host range of NS alleles between the avian species is not strict, which is consistent with previous studies showing infection and persistence of both NS alleles A and B viruses in poultry [41,48]. In addition, NS allele A and B showed comparable shedding patterns and routes for most subtypes, except H8N4, of which the NS A variant was shed more efficiently than the NS B variant. In tissue samples, the detection of virus was variable between strains, which appeared to be independent of NS allele. The results thus indicate variable replication efficiencies of NS A and B viruses in chickens.

To study the innate immune response to the different LPAI viruses, we measured changes in the production of inflammatory cytokines in the respiratory and intestinal tract of the virus-inoculated chickens. We measured the levels of mRNA encoding for interleukins IL-6 and IL-1β and interferons IFN-α and IFN-β, which can inhibit viral replication by interaction with viral components and modulation of the host cell metabolism [43], and TLR-7, which is an endosomal pattern recognition receptors that can detect viral RNA at the site of infection to induce an inflammatory response [49]. In the trachea, no stimulation of the innate immune response was observed at 1 dpi, despite virus was detected in all virus-inoculated groups. At 3 dpi, IFN-β mRNAs were induced to higher levels in response to NS A viruses of both wild bird-associated and poultry-associated subtypes. The upregulation of IFN-β mRNA expression has previously been observed in LPAI virus-infected chickens [21]. However, the results do not correspond with the expected inhibition of IFN type I production by NS A viruses. In the ileum, no induction of cytokine mRNA expression was observed at 5 dpi. The expression of IFN-α mRNA was even slightly reduced in some groups. At 7 dpi, the expression of IL-1β, IFN-α, IFN-β and TLR7 mRNAs was induced by LPAI viruses of wild bird-associated subtypes carrying NS allele B in the ileum of some, but not all, chickens, indicating that immune responses vary between strains. The induction of inflammatory cytokines in the respiratory and intestinal tract has been reported in previous studies using LPAI viruses of subtypes H7N1 [21] and H9N2 [50], but is limited compared to HPAI viruses [43]. Our study also indicates that LPAI viruses elicit limited innate immune responses in chickens. The cytokine response remained limited to undetectable when only virus-positive samples were included [51]. It should be mentioned that the analysis of the cytokine response was limited to few time points and tissues. In addition, the involvement of other (non-studied) cytokines in limiting LPAI virus infection cannot be excluded.

The susceptibility of chickens to the viruses used in this infection experiment did not correlate with the detection frequencies of the HA and NA subtypes or NS alleles in the field. None of the poultry-associated aa residues, as specified in Table S2, were present in the inoculated viruses, except the K391R substitution in the PA protein of H3N8 NS B virus and both H4N6 and H9N2 viruses. In an attempt to explain the unexpected results, we also analyzed potential links between the internal genes of the inoculated viruses and previous isolations of similar genes from poultry hosts. Although the number of poultry viruses in the genetic clusters was often limited, this analysis suggested a potential role for the PB1, PB2, and PA genes in determining infectivity of these virus strains in chickens. Host-restriction factors in the polymerase genes have been described previously in several studies on avian-human transmission of AI viruses [52,53]. Also, previous proteotype analysis has shown specific combinations of the viral proteins among AI viruses of human and avian origin [54], Our study also indicates that a set of proteins may be the major host range determinant. However, the role of the polymerase proteins in transmission between avian hosts is poorly understood. Additional studies should be conducted in order to investigate the contribution of the polymerase activities in determining host range of AI viruses among avian species.

Concluding, this study provides valuable information on the susceptibility of chickens to LPAI viruses of various subtypes and genotypes. It demonstrates that wild bird–associated LPAI viruses of subtypes H3N8 and H4N6 can readily replicate in experimentally infected chickens, despite their infrequent detection in Dutch poultry flocks. The variable susceptibility of chickens to poultry-associated subtypes and NS alleles could not be explained by differences in the innate immune response, which was limited in all chickens. The results in this study increase our understanding of LPAI virus infection dynamics in chickens and can be used to optimize surveillance strategies for LPAI viruses in wild birds and poultry.

## Figures and Tables

**Table 1:** Viral replication in the trachea, lung and ileum. The ratio of chickens virus-positive in tissue to the number of virus-inoculated chickens. The tissues were collected from four euthanized birds of each experimental group at 1, 3, 5, and 7 days post inoculation (dpi). Virus was detected by influenza virus-specific PCR (M-PCR). Viral titres are expressed as the mean Ct value ± standard deviation (SD).

Virus	Trachea		Lung		Ileum		
	1 dpi	3 dpi	1 dpi	5 dpi	1 dpi	5 dpi	7 dpi
H3N8 NS allele A	3/4 (32.6 ± 1.8)	1/4 (34.3)	4/4 (31.6 ± 3.4)	0/4	0/4	0/4	1/4 (26.3)
H3N8 NS allele B	1/4 (29.5)	0/4	4/4 (34.6 ± 2.8)	0/4	0/4	1/4 (23.2)	2/4 (23.4 ± 0.2)
H4N6 NS allele A	3/4 (27.5 ± 0.8)	0/4	3/4 (26.9 ± 2.9)	0/4	0/4	0/4	0/4
H4N6 NS allele B	1/4 (36.6)	0/4	3/4 (32.5 ± 8.3)	0/4	0/4	0/4	0/4
H8N4 NS allele A	2/4 (32.3 ± 0.2)	0/4	4/4 (30.2 ± 0.5)	0/4	0/4	0/4	4/4 (34.2 ± 4.9)
H8N4 NS allele B	0/4	0/4	1/4 (35.7)	1/4 (30.0)	0/4	0/4	0/4
H9N2 NS allele A	2/4 (30.9 ± 0.9)	0/4	0/4	0/4	0/4	0/4	0/4
H9N2 NS allele B	2/4 (30.1 ± 2.3)	0/4	2/4 (33.5 ± 2.0)	0/4	0/4	0/4	0/4

dpi, days post inoculation; NS, nonstructural protein; n.d., not done.

**Table 2:** Genetic cluster analysis of the inoculated viruses. Clusters of internal genes similar to those of the inoculated viruses, presented as the number of sequences that originate from poultry out of the total number of sequences within each cluster (cluster size). Genetic clusters were generated by clustering publicly available sequences of around 20,000 avian-origin AI viruses obtained from GISAID’s EpiFlu database (http://www.gisaid.org) [26] on 27 May 2019 against the sequences of the inoculated viruses, using a sequence identity threshold value of 1.5%. We gratefully acknowledge the authors, originating and submitting laboratories of the sequences from GISAID’s EpiFlu Database on which this research is based.

Virus	PB1	PB2	PA	NP	MP	NS
H3N8 NS allele A	3/15	0/91	0/23	2/37	0/49	0/67
H3N8 NS allele B	0/4	2/6	0/7	0/32	7/101	0/103
H4N6 NS allele A	4/17	0/59	1/5	1/27	6/304	13/557
H4N6 NS allele B	6/17	0/97	0/3	0/95	34/568	0/108
H8N4 NS allele A	0/160	2/33	0/21	0/92	38/698	33/886
H8N4 NS allele B	0/61	0/20	0/20	4/56	93/1034	0/79
H9N2 NS allele A	0/59	0/64	0/58	0/108	14/510	2/86
H9N2 NS allele B	0/15	0/16	0/56	0/1	0/149	8/35

PB2, polymerase basic protein 2; PB1, polymerase basic protein 1; PA, polymerase acidic protein; HA, hemagglutinin; NP, nucleoprotein; NA, neuraminidase; MP, matrix protein; NS, nonstructural protein.

## Supplementary Materials

The following are available online at https://www.mdpi.com/1999-4915/11/11/1010/s1, Table S1. Viruses used in the infection experiment. Table S2. Meta-CATS analysis for virus selection. Table S3. Influenza virus–specific antibody detection. Table S4. Oropharyngeal shedding. Table S5. Cloacal shedding.
