# Supplementary material for: Susceptibility of Chickens to Low Pathogenic Avian Influenza (LPAI) Viruses of Wild Bird– and Poultry–Associated Subtypes
Source: Viruses. 2019 Oct 31;11(11):1010. doi: 10.3390/v11111010 (PMC6893415; doi:10.3390/v11111010)
Supplement: Supplementary file 1 [file viruses-11-01010-s001.zip › Table S1.pdf]

Table S1. Viruses used in the infection experiment. Low pathogenic avian influenza (LPAI) viruses used in this study, including the isolate identification numbers as listed in GISAID's EpiFlu database (http://www.gisaid.org). The viruses were collected from wild ducks as part of the national avian influenza (AI) virus surveillance program in wild birds in the Netherlands.

**Virus**	**Isolate name**	**GISAID isolate ID**
H3N8 NS allele A	A/Mallard/Netherlands/17/2011	EPI_ISL_243527
H3N8 NS allele B	A/Mallard/Netherlands/30/2010	EPI_ISL_243634
H4N6 NS allele A	A/Mallard/Netherlands/7/2011	EPI_ISL_243399
H4N6 NS allele B	A/Mallard/Netherlands/26/2010	EPI_ISL_243498
H8N4 NS allele A	A/Mallard/Netherlands/1/2006	EPI_ISL_33854
H8N4 NS allele B	A/Mallard/Netherlands/11/2006	EPI_ISL_267765
H9N2 NS allele A	A/Mallard/Netherlands/19/2005	EPI_ISL_267193
H9N2 NS allele B	A/Eurasian wigeon/Netherlands/3/2005	EPI_ISL_64643
