# Supplementary material for: Susceptibility of Chickens to Low Pathogenic Avian Influenza (LPAI) Viruses of Wild Bird– and Poultry–Associated Subtypes
Source: Viruses. 2019 Oct 31;11(11):1010. doi: 10.3390/v11111010 (PMC6893415; doi:10.3390/v11111010)
Supplement: Supplementary file 1 [file viruses-11-01010-s001.zip › Table S2_revised.pdf]

Table S2. Meta-CATS analysis for identification of host-associations in the internal proteins. A statistical comparative analysis was performed on 162 wild bird low pathogenic avian influenza (LPAI) viruses and 42 poultry LPAI viruses using the metadata-driven comparative analysis tool for sequences (meta-CATS) of the Influenza Research Database (IRD) (https://www.fludb.org). In the statistical tool, a chi-square test of independence was performed at each amino acid (aa) position to identify residues that significantly differed between the host groups ($p<0.05$). Subsequently, significant aa positions identified by meta-CATS that were present in 90% of the viruses in both host groups, or showed high variability (≥ 3 aa variants), were excluded. The remaining significant aa positions are shown below, including the percentage of wild bird and poultry viruses carrying the different aa variants. The virus sequences used in this study were generated in a previous study (for details see Material and Methods).

Segment	aa position	aa variant	% wild bird viruses	% poultry viruses	p-value
PB1	577	K	97	80	<0.01
		R	3	20	
PB2	255	V	98	88	<0.05
		I	2	12	
PA	391	R	83	98	<0.05
		K	17	2	
NP	77	K	100	80	<0.01
		R	0	20	
M2	18	K	97	80	<0.01
		R	3	20	
NS	82 different positions	allele A	62	83	<0.01
		allele B	38	17	

PB2, polymerase basic protein 2; PB1, polymerase basic protein 1; PA, polymerase acidic protein; HA, hemagglutinin; NP, nucleoprotein; NA, neuraminidase; M1, matrix protein 1; M2, matrix protein 2; NS1, nonstructural protein 1; NS2, nonstructural protein 2.
