# Supplementary material for: Susceptibility of Chickens to Low Pathogenic Avian Influenza (LPAI) Viruses of Wild Bird– and Poultry–Associated Subtypes
Source: Viruses. 2019 Oct 31;11(11):1010. doi: 10.3390/v11111010 (PMC6893415; doi:10.3390/v11111010)
Supplement: Supplementary file 1 [file viruses-11-01010-s001.zip › Table S3.pdf]

**Table S3. Influenza virus-specific antibody detection.** The ratio of antibody-positive chickens to the number of virus-inoculated chickens as determined by influenza-specific ELISA and subtype-specific hemagglutinin inhibition (HI) tests. Serum samples taken at 5 and 7 days post inoculation (dpi) are shown. The HI titres are expressed as the mean $\log_2$ values ± standard deviation (SD).

| | **ELISA positives** | | **HI test positives (titre)** [a] | |
|---|---|---|---|---|
| **Virus group** | **5 dpi** | **7 dpi** | **5 dpi** | **7 dpi** |
| H3N8 NS allele A | 0/4 | 2/4 | 0/4 | 1/4 (3) |
| H3N8 NS allele B | 0/4 | 2/4 | 0/4 | 1/4 (3) |
| H4N6 NS allele A | 0/4 | 1/4 | 0/4 | 0/4 |
| H4N6 NS allele B | 0/4 | 4/4 | 0/4 | 0/4 |
| H8N4 NS allele A | 4/4 | 3/4 | 3/4 (4.3±0.6) | 2/4 (5.0±0.0) |
| H8N4 NS allele B | 0/4 | 0/4 | 0/4 | 0/4 |
| H9N2 NS allele A | 1/4 | 3/4 | 0/4 | 0/4 |
| H9N2 NS allele B | 0/4 | 1/4 | 0/4 | 0/4 |

dpi, days post inoculation; HI, hemagglutination inhibition; NS, nonstructural protein
[a] HI titres of 3 $\log^2$ or higher were considered positive.
