# Supplementary material for: Susceptibility of Chickens to Low Pathogenic Avian Influenza (LPAI) Viruses of Wild Bird– and Poultry–Associated Subtypes
Source: Viruses. 2019 Oct 31;11(11):1010. doi: 10.3390/v11111010 (PMC6893415; doi:10.3390/v11111010)
Supplement: Supplementary file 1 [file viruses-11-01010-s001.zip › Table S4_revised.pdf]

Table S4. Oropharyngeal shedding. The ratio of chickens positive for viral shedding through the oropharyngeal (OP) route to the number of virus-inoculated chickens. The chickens inoculated by the intranasal (IN) and intratracheal (IT) route with eight strains of low pathogenic avian influenza (LPAI) viruses ($10^{5.3}$ median egg infectious dose (EID_{50}) per bird). The swabs were taken daily from live birds to 7 days post inoculation (dpi) for virus detection by influenza virus-specific PCR (M-PCR). Viral titres for positive samples are expressed as the mean equivalent log10 EID_{50}/ml titre ± standard deviation (SD). The onset of viral shedding was calculated based on the positive swabs and is reported as mean dpi ± SD.

Virus group	0 dpi	1 dpi	2 dpi	3 dpi	4 dpi	5 dpi	6 dpi	7 dpi	Total	Onset of viral shedding (dpi)
H3N8 NS allele A	0/20	9/20 (4.0±1.2)	6/16 (3.7±0.8)	4/12 (2.7±0.4)	3/8 (3.1±0.8)	7/8 (3.0±0.8)	2/4 (2.5±0.1)	1/4 (2.3)	12/20 (3.6±0.9)	1.8±1.4
H3N8 NS allele B	0/20	13/20 (4.7±1.2)	9/16 (4.8±1.1)	4/12 (4.1±1.4)	4/8 (4.2±1.5)	0/8	0/4	0/4	17/20 (4.6±1.2)	1.2±0.5
H4N6 NS allele A	0/20	15/20 (4.8±1.1)	9/16 (3.8±0.9)	0/12	1/8 (2.8)	1/8 (3.0)	1/4 (3.0)	0/4	19/20 (4.3±1.1)	1.5±1.4
H4N6 NS allele B	0/20	15/20 (4.7±1.2)	12/16 (4.9±0.8)	5/12 (4.0±1.2)	4/8 (4.6±0.7)	3/8 (4.2±0.6)	1/4 (5.2)	0/4	16/20 (4.7±1.0)	1.1±0.3
H8N4 NS allele A	0/20	5/20 (2.4±0.9)	0/16	0/12	0/8	1/8 (1.9)	1/4 (1.8)	0/4	7/20 (2.5±0.9)	2.3±2.2
H8N4 NS allele B	0/20	0/20	0/16	0/12	0/8	0/8	0/4	0/4	0/20	na
H9N2 NS allele A	0/20	1/20 (2.7)	1/16 (1.9)	0/12	0/8	0/8	0/4	0/4	4/20 (2.3±0.5)	1.3±0.5
H9N2 NS allele B	0/20	4/20 (2.5±0.4)	1/16 (3.1)	1/12 (2.4)	0/8	0/8	0/4	0/4	6/20 (2.6±0.4)	1.2±0.4

OP, oropharyngeal; dpi, days post inoculation; NS, nonstructural protein
