# Supplementary material for: Susceptibility of Chickens to Low Pathogenic Avian Influenza (LPAI) Viruses of Wild Bird– and Poultry–Associated Subtypes
Source: Viruses. 2019 Oct 31;11(11):1010. doi: 10.3390/v11111010 (PMC6893415; doi:10.3390/v11111010)
Supplement: Supplementary file 1 [file viruses-11-01010-s001.zip › Table S5_revised.pdf]

Table S5. Cloacal shedding. The ratio of chickens positive for viral shedding through the cloacal (CL) route to the number of virus-inoculated chickens. The chickens inoculated by the intranasal (IN) and intratracheal (IT) route with eight strains of low pathogenic avian influenza (LPAI) viruses ($10^{5.3}$ median egg infectious dose (EID_{50}) per bird). The swabs were taken daily from live birds to 7 days post inoculation (dpi) for virus detection by influenza virus-specific PCR (M-PCR). Viral titres for positive samples are expressed as the mean equivalent log10 EID_{50}/ml titre ± standard deviation (SD). The onset of viral shedding was calculated based on the positive swabs and is reported as mean dpi ± SD.

Virus group	0 dpi	1 dpi	2 dpi	3 dpi	4 dpi	5 dpi	6 dpi	7 dpi	Total	Onset of viral shedding (dpi)
H3N8 NS allele A	0/20	0/20	1/16 (6.3)	1/12 (6.6)	2/8 (4.9±2.7)	1/8 (6.2)	1/4 (4.8)	2/4 (3.7±1.0)	2/20 (5.1±1.6)	3.0±1.4
H3N8 NS allele B	0/20	0/20	1/16 (7.9)	3/12 (4.7±2.4)	2/8 (6.1±0.3)	2/8 (5.9±0.6)	2/4 (4.7±2.3)	2/4 (6.8±0.4)	7/20 (6.0±1.3)	4.1±1.8
H4N6 NS allele A	0/20	0/20	0/16	0/12	0/8	0/8	0/4	0/4	0/20	na
H4N6 NS allele B	0/20	0/20	0/16	0/12	0/8	0/8	0/4	0/4	0/20	na
H8N4 NS allele A	0/20	0/20	0/16	1/12 (4.9)	2/8 (3.5±1.1)	2/8 (5.5±0.2)	2/4 (4.6±0.3)	2/4 (4.9±0.6)	3/20 (4.7±0.9)	4.0±1.0
H8N4 NS allele B	0/20	0/20	0/16	0/12	0/8	0/8	0/4	0/4	0/20	na
H9N2 NS allele A	0/20	0/20	0/16	0/12	0/8	0/8	0/4	0/4	0/20	na
H9N2 NS allele B	0/20	0/20	0/16	0/12	0/8	0/8	0/4	0/4	0/20	na

CL, cloacal; dpi, days post inoculation; NS, nonstructural protein
